# CO_2_ Separation in Nanocomposite Membranes by the Addition of Amidine and Lactamide Functionalized POSS^®^ Nanoparticles into a PVA Layer

**DOI:** 10.3390/membranes8020028

**Published:** 2018-06-08

**Authors:** Gabriel Guerrero, May-Britt Hägg, Christian Simon, Thijs Peters, Nicolas Rival, Christelle Denonville

**Affiliations:** 1 Department of Chemical Engineering, Norwegian University of Science and Technology, 7491 Trondheim, Norway; gabriel.g.heredia@ntnu.no; 2 SINTEF Industry, 0314 Oslo, Norway; christian.r.simon@sintef.no (C.S.); Thijs.peters@sintef.no (T.P.); nicolas.rival@sintef.no (N.R.); christelle.denonville@sintef.no (C.D.)

**Keywords:** POSS^®^, nanocomposite membranes, CO_2_ separation, PVA

## Abstract

In this article, we studied two different types of polyhedral oligomeric silsesquioxanes (POSS^®^) functionalized nanoparticles as additives for nanocomposite membranes for CO_2_ separation. One with amidine functionalization (Amidino POSS^®^) and the second with amine and lactamide groups functionalization (Lactamide POSS^®^). Composite membranes were produced by casting a polyvinyl alcohol (PVA) layer, containing either amidine or lactamide functionalized POSS^®^ nanoparticles, on a polysulfone (PSf) porous support. FTIR characterization shows a good compatibility between the nanoparticles and the polymer. Differential scanning calorimetry (DSC) and the dynamic mechanical analysis (DMA) show an increment of the crystalline regions. Both the degree of crystallinity ($X_{c}$) and the alpha star transition, associated with the slippage between crystallites, increase with the content of nanoparticles in the PVA selective layer. These crystalline regions were affected by the conformation of the polymer chains, decreasing the gas separation performance. Moreover, lactamide POSS^®^ shows a higher interaction with PVA, inducing lower values in the CO_2_ flux. We have concluded that the interaction of the POSS^®^ nanoparticles increased the crystallinity of the composite membranes, thereby playing an important role in the gas separation performance. Moreover, these nanocomposite membranes did not show separation according to a facilitated transport mechanism as expected, based on their functionalized amino-groups, thus, solution-diffusion was the main mechanism responsible for the transport phenomena.

## 1. Introduction

Global warming is one of the major problems that face humanity. Global warming is caused by the emission of greenhouse gases, of which the main component is carbon dioxide (CO_2_). CO_2_ emissions have dramatically increased in the last 50 years, and are still continually increasing each year [1]. The burning of fossil fuels contributes to most of the CO_2_ emissions, and hence, there is a significant interest in developing technologies to reduce CO_2_ emissions. Carbon capture and storage (CCS) has become an important instrument to reach the goals agreed on the 2015 Paris international convention [2]. Membrane technologies have seen an important growth in this market [3,4,5,6]. However, its application in large CO_2_ capture processes in the power and industrial sector has not yet matured. Key challenges are related to the low partial pressure of CO_2_ and the large scale required for flue gas treatment. For membranes to be cost-effective, further innovations in process design and membrane materials are thus needed. In this respect, the use of hybrid composite membranes that benefit from both an organic and inorganic part to further improve the gas separation performance have been widely studied. Membrane-based separation processes are not only cost effective and environmentally friendly, but also offer much more versatility and simplicity for customizing system designs with many novel polymeric materials now available. Moreover, membrane modularity and easy scale-up opens for retrofitting existing plants, as well as flexibility with respect to the CO_2_ capture rate. The technology has, as well, a near instantaneous response, and a high turndown is possible that preserves plant operability.

The ability to selectively remove one component in a mixture while rejecting others describes the perfect separation device [7]. Various materials and methods are being developed for capture and storage, and in some cases, conversion, to mitigate the effect of global warming. Emerging and already established concepts for CO_2_ capture encounter the challenge of finding an economically feasible and efficient separation technology from effluent gas streams [8,9]. The study of advanced materials and modern manufacturing methods have helped to obtain new and improved membranes that have better separation performances, thus contributing to obtain environmental friendly gas membrane separation processes which demand less energy. In this way, due to the low cost and easy processability of polymeric membranes, they have, so far, been a promising alternative for the non-condensable gas separation. Today, only a few polymer membrane materials are being used in industrial applications. However, in research, various polymers are being studied and reported to be potential candidates for use on commercial scale, due to good results achieved on lab scale [10]. Membranes require a high gas permeation rate combined with a high selectivity for process applications. Incrementing the performance of the membrane by increasing the permeance, will result in requiring less membrane area, which will help to reduce the costs and environmental imprint. Variables, such as mechanical and thermal stability of the material at the operating conditions, should also be considered when selecting the membrane material. The proper selection of a highly stable material, with high performance, has made the effort complex, and the research for an optimum material is continuously in progress [11,12,13,14,15]. 

The recognized trade-off between gas permeability and selectivity has resulted in a rather slow development of new polymeric membranes [16]. Nevertheless, both parameters need to be considered to have a promising membrane for industrial processes. In this case, the use of novel hybrid materials for making membranes that incorporate both an organic and an inorganic part, have opened new opportunities to develop new materials, and hence, bypass the limits that exist today. Both, the organic part and the inorganic part will, in different ways, contribute to the transport of gases through the hybrid membrane, while additionally, the inorganic part may contribute to mechanical and thermal strength. A lot of research has been published on the addition of zeolites to polymers for gas separation as mixed matrix membranes (MMMs) [17,18,19]. For instance, ZIF-71 nanoparticles, embedded into PEOT/PBT copolymer, have shown capability for dehumidification and CO_2_ separation [20] in industrial use, and polymers of intrinsic microporosity (PIMs) are used to obtain membranes with high gas permeability [21].

On the other hand, facilitated transport membranes (FTMs) use a carrier to increase the transport of the desired gas through a membrane, without having a negative effect on the selectivity. Many researchers have studied this type of membrane [5,22,23,24,25]. 

In this study, facilitated transport was expected for CO_2_ based on the selected nanoparticles. The manufactured membranes involve the addition of carriers that are expected to improve the gas separation properties. Nanosized particles, such as polyhedral oligomeric silsesquioxanes (POSS^®^) embedded in a polymer matrix, are reported here. POSS^®^ nanoparticles have a rigid cage-like structure which is an intermediate between silica and siloxane. POSS^®^ materials are well-defined, three-dimensional nano building blocks that can contribute to create unique hybrid materials, with a precise control of nanostructure and properties [26]. POSS^®^ chemical reagents are nanostructured, with sizes of 1–3 nm. However, unlike silica or silicones, POSS^®^ can be produced with nonreactive substituents, to make them more or less compatible with different monomers or polymers, depending on the way they are obtained [27]. The amino and amidino groups of the chosen POSS^®^ were expected to provide the suitable carriers for CO_2_ when humidity is present. This feature makes POSS^®^ an attractive candidate for the fabrication of hybrid materials. POSS^®^ has been used in several gas separation studies in recent years [28,29,30,31].

In our previous work [32], we have tested amino POSS^®^ without achieving any improvement in CO_2_ separation. In our effort to increase the performance of the membranes, we have modified these amino nanoparticles into the amidino and lactamide particles reported here. The aim of the current work reported here is to investigate the effect on the gas separation performance of the membranes by the addition of two different functionalized POSS^®^ nanoparticles; one with amidine groups (Amidino POSS^®^) and the other with amino and lactamide functional groups (Lactamide POSS^®^). Amino groups are versatile and can undergo different chemical reactions. The functionalization of POSS^®^ can thus be tailor-made, depending on the aimed properties. Amidino POSS^®^ has been specially developed to enhance CO_2_ capture capacity. Amidino POSS^®^ and Lactamide POSS^®^ would be expected to exhibit different mechanisms toward CO_2_ capture. While Lactamide POSS^®^ would catch CO_2_ through forming a competition between carbonate and carbamate, Amidino POSS^®^ would only form carbonate which is beneficial in term of cyclic capacity [32]. Moreover, functionalization of aminopropyl POSS^®^ with lactic moieties is believed to improve compatibility of Lactamide POSS^®^ with PVA, as it contains secondary hydroxyl groups.

## 2. Materials and Methods 

### 2.1. Materials

**Figure 1:**
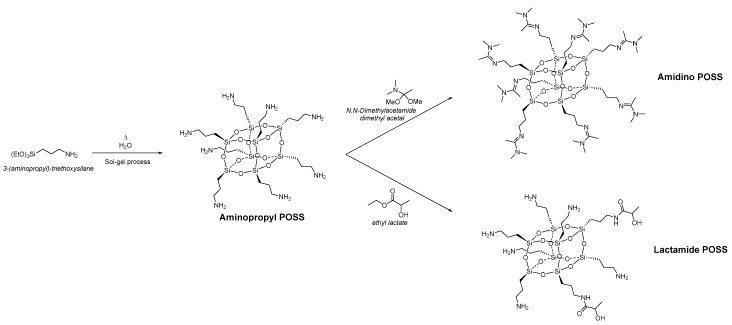
Synthesis of Amidino POSS^®^ and Lactamide POSS^®^, respectively.

Polyvinyl alcohol (PVA) was provided by Sigma-Aldrich, the molecular weight of 85,000–124,000, 87–89% hydrolyzed. Polysulfone (PSf) porous support flat sheet membranes with a molecular weight cut-off 50,000 Da were purchased from Alfa Laval (Denmark). The PSf support was washed. Two different types of nanoparticles belonging to the class of the polyhedral oligomeric silsesquioxane (POSS^®^) were synthesized. Figure 1 shows the synthesis of the two different types and their functionalization. The aminopropyl POSS^®^ was synthesized by classical sol-gel synthesis from the 3-(aminopropyl)-triethoxysilane. Aminopropyl POSS^®^ particles were subsequently functionalized in solvent-free reactions with *N*,*N*-dimethylacetamide dimethyl acetal to obtain Amidino POSS^®^ and with ethyl lactate to prepare Lactamide POSS^®^. Amidino POSS^®^ and Lactamide POSS^®^ are obtained in *n*-propanol with a solid content of 42% for Amidino POSS^®^ and 32% for Lactamide POSS^®^.

### 2.2. Membrane Preparation

PVA (3.0 g) was added to distilled water (97 g). The mixture was refluxed for two hours to dissolve the polymer under stirring at 500 rpm at 90 °C. Then, the PVA solution was cooled down to room temperature. Amidino POSS^®^ and Lactamide POSS^®^ solutions were stabilized separately, by stirring at 500 rpm for at least 15 min, before being mixed with the PVA solution. These nanoparticle solutions were then added dropwise to the PVA solution by filtering through a PTFE 5 µm syringe filter according to the required POSS^®^/PVA ratio. Afterwards, the solution was filtered again through a 5 µm PTFE syringe filter, and subsequently stirred for at least 30 min. Five centimeter diameter membranes of PSf were cut. The flat sheet composite membranes were prepared by casting 1 mL of the solution over the PSf disc. They were air dried overnight at 45 °C, and further air dried at 100 °C for one hour before a natural cooling, until the oven had reached 40 °C. These flat sheet composite membranes were used for SEM morphology characterization and gas permeation performance evaluation. The self-supported membrane samples for DSC, DMA, and FTIR characterization were prepared following the same temperature profile by using 5 mL of the POSS^®^/PVA solution in a glass petri dish.

### 2.3. Membrane Characterization

#### 2.3.1. Scanning Electron Microscopy (SEM)

The thickness and surface of the composite membranes were studied using a Hitachi TM3030 and a Nova NanoSEM650 (FEI corp., Tokyo, Japan) field emission gun scanning electron microscope (FEG-SEM, Tokyo, Japan). The cross-section of the samples was prepared by freeze-fracturing/cutting in liquid nitrogen. The samples were sputter coated with a thin gold layer (90 s) to provide electronic conductivity to the samples.

#### 2.3.2. Differential Scanning Calorimetry (DSC)

The thermal properties of the membrane materials were investigated using a differential scanning calorimeter (DSC 214, Netzsch, Selb, Germany). A sample of about 10–12 mg was placed in an aluminum pan covered with a proper lid, together with a standard empty pan, into the DSC sample holder. To eliminate the thermal history and remove any residual solvent, the samples were first heated from room temperature to 110 °C at 10 K/min, kept at this temperature for 10 min, and cooled to −50 °C at a rate of 10 K/min. The samples were then heated up from room temperature to 300 °C at a heating rate of 10 K/min, under an N_2_ atmosphere. The analysis was then carried out from the second heating scan.

#### 2.3.3. Thermogravimetric Analysis (TGA)

The thermal stability of the membranes was investigated with a thermogravimetric analyzer (TG209 F1, Netzsch, Selb, Germany). Around 10–12 mg of sample was placed in the sample pan and heated from 30 °C to 800 °C (10 K/min). Nitrogen was used as both the balance and sweep gas, with flow rates of 20 mL/min.

#### 2.3.4. Fourier Transform Infrared Spectroscopy (FTIR)

FTIR was used to investigate the possible interaction between POSS^®^ nanoparticles with the composite membranes. The IR spectroscopy experiments were carried out with a FTIR spectrometer (iS50 FT-IR, Nicolet, Thermo Fisher Scientific, Waltham, MA, USA) with a smart endurance reflection cell. 

#### 2.3.5. Dynamic Mechanical Analysis (DMA)

Dynamic mechanical analysis was conducted on a DMA 242 E Artemis (Netzsch, Selb, Germany) in tension mode under the following conditions: frequency 1 Hz, a proportional factor of 1.1, absolute target amplitude of 40 µm, a maximum dynamic force of 2.182 N, and no additional static force. To eliminate the thermal history, the samples were first heated from room temperature to 80 °C at 5 K/min, kept at this temperature for 10 min, and then cooled afterwards to −50 °C at a rate of 2 K/min, to be heated up again to 350 °C at a rate of 2 K/min. The samples were cut with 10 mm length and 5 mm width, with a thickness between 0.020–0.060 mm. With DMA, various transition temperatures can be determined while the mechanical properties are measured under an oscillatory strain, which supplies information about major transitions, as well as secondary and tertiary transitions not identifiable by other methods [33].

#### 2.3.6. Gas Permeation Performance Evaluation

The membrane performance was measured in a feed gas mixture at a pressure of 1.3, 2, and 3 bars with a mixed gas permeation test rig, described elsewhere [34]. The experimental specifications, such as flow set parameters, temperature, and gas composition in feed and sweep streams, can be found in our previous work [32]. The CO_2_ flux and CO_2_/N_2_ selectivity were calculated from the measured permeate CO_2_ and N_2_ concentration in the sweep flow gas. 

The mixed gas permeability of CO_2_ and N_2_ was calculated assuming perfect mixing through Equation (1).

(1) $$P_{A}=\frac{J_{A}\;\cdot \;\ell }{x_{fA\;}\cdot \;P_{f}-x_{pA\;}\cdot \;P_{p}}$$

where $P_{A}$ represents the permeability (Barrer) of component A (CO_2_ or N_2_), $J_{A}$ is the flux (m^3^(STP)/m^2^·h), $x_{fA}$ and $x_{pA}$, represent molar fraction of the component on feed and permeate side respectively, $P_{f}$ and $P_{p}$ (bar) are the absolute pressure on feed and permeate side, and ℓ (µm) is the selective measured layer thickness. 

The CO_2_/N_2_ selectivity $\alpha_{A/B}$, which corresponds to the ratio of the permeabilities for gases A (CO_2_) and B (N_2_), is given as follows:

(2) αAB=PAPB.

## 3. Result and Discussion

### 3.1. Morphology of the Membranes 

#### 3.1.1. SEM

**Figure 2:**
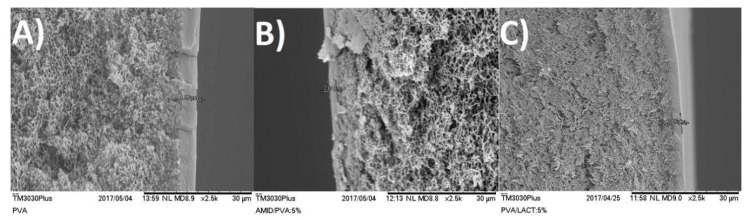
SEM-SE cross section pictures from the PSf support flat sheet membranes (**A**) PVA; (**B**) Amidino POSS^®^/PVA:5%; (**C**) Lactamide POSS^®^/PVA: 5%.

**Figure 3:**
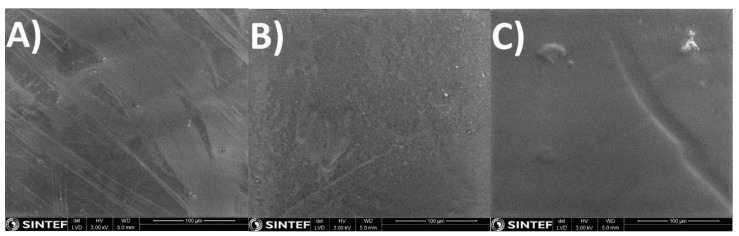
SEM-SE surface pictures from the PSf support flat sheet membranes (**A**) PVA; (**B**) Amidino POSS^®^/PVA: 5%, Lactamide POSS^®^/PVA: (**C**) 5%.

The composite dense layer deposited on the porous support can be seen in Figure 2 and Figure 3, where examples are given for PVA, Amidino POSS^®^/PVA: 5%, and Lactamide POSS^®^/PVA: 5%. The dense composite membranes have an average thickness of 1–4 µm. The correct thickness of each membrane has been taken into account when the permeability is calculated. After scanning several membranes and places of the membrane, we conclude that a continuous composite PVA layer is formed on the support, and no evidence of pores or defects was observed. The white points in Figure 3A–C are most likely dust coming from the preparation of the SEM sample.

#### 3.1.2. FTIR

**Figure 4:**
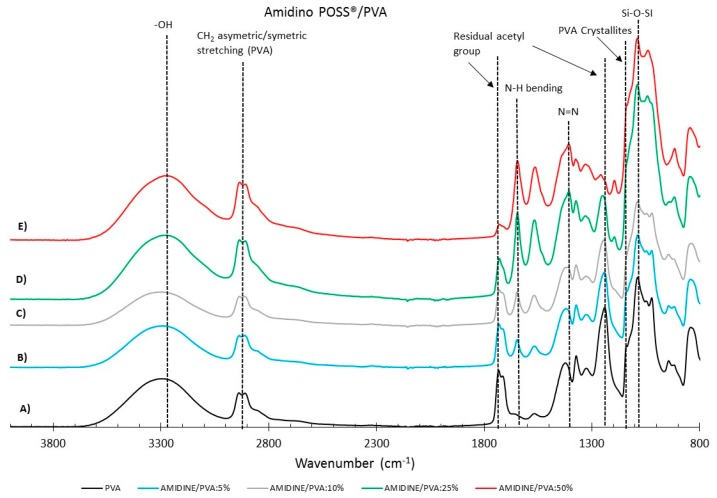
FTIR reflectance of (A) pure PVA, Amidino POSS^®^/PVA: (B) 5%; (C) 10%; (D) 25%; (E) 50%. The curves are shifted vertically for clarity.

**Figure 5:**
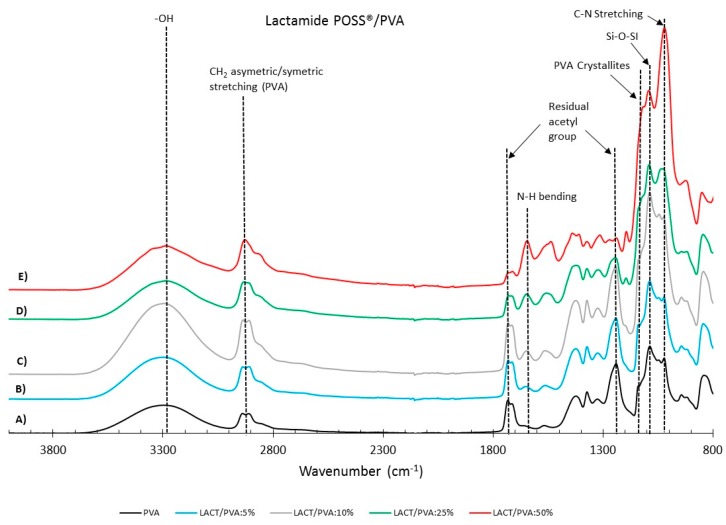
FTIR reflectance of (A) pure PVA, Lactamide POSS^®^/PVA: (B) 5%; (C)10%; (D) 25%; (E) 50%. The curves are shifted vertically for clarity.

Figure 4 and Figure 5 show the FTIR spectra for the Amidino POSS^®^/PVA and Lactamide POSS^®^/PVA systems, respectively. The main IR peaks are given in Table 1. The characteristic peaks of PVA are seen at 1142, 1720, 2850, and 3260 cm^−1^, the peaks for residual acetyl groups 1740, 1265 cm^−1^ decrease with addition of nanoparticles, since ratio PVA/nanoparticle loading is increasing, and thus, less PVA is present, and the residual acetyl peak decreases. The other peaks can be ascribed to either Amidino or Lactamide POSS^®^ functionalities. The peak in the range of 1080 cm^−1^ corresponds to the asymmetric (Si–O–Si) stretching vibration band, belonging to the silica cage of POSS^®^. Its intensity increases with the nanoparticle loading [35]. 

The absorption peak at approximately 1142 cm^−1^ corresponds to the PVA crystallites [36], increasing with the nanoparticle concentration. This is due to the interaction of the nanoparticles that work as a nucleating agent, which is more deeply explained in Section 3.2.2. The range at about 1400 cm^−1^ belongs to the azo compound N=N stretching of the Amidino POSS^®^ nanoparticles, and shows a visible increment for the spectra of 25 and 50% ratio of nanoparticles. Finally, the range between 1550–1650 cm^−1^ belongs to the secondary amides bending, that also increases with the addition of nanoparticles. The Lactamide POSS^®^/PVA system shows the aliphatic stretch for C–N at 1020 cm^−1^, the presence of POSS^®^ at 1080 cm^−1^, and the secondary amines bending, that increases with the addition of nanoparticles between 1550–1650 cm^−1^. These spectra confirm the interaction between POSS^®^ nanoparticles with the polymer in the composite membranes, which is influenced by the loading concentration of nanoparticles.

### 3.2. Thermomechanical Properties 

#### 3.2.1. Thermogravimetric Analysis (TGA)

**Figure 6:**
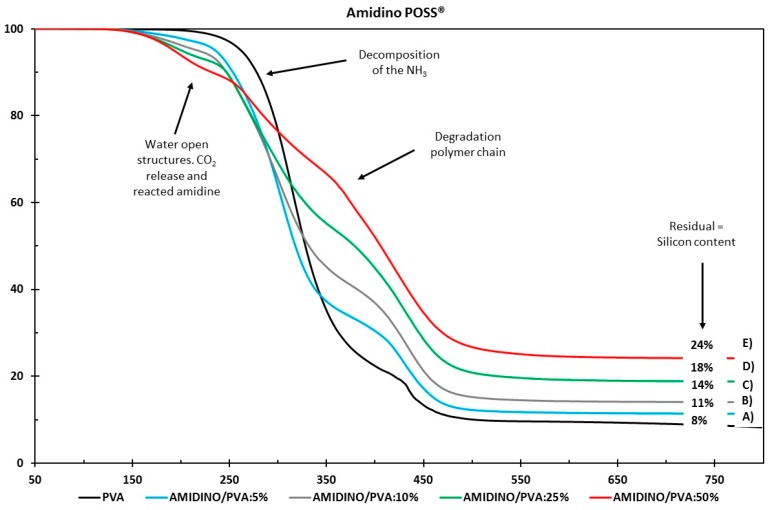
Thermogravimetric analysis of (A) PVA, Amidino POSS^®^/PVA: (B) 5%; (C) 10%; (D) 25%; (E) 50%.

**Figure 7:**
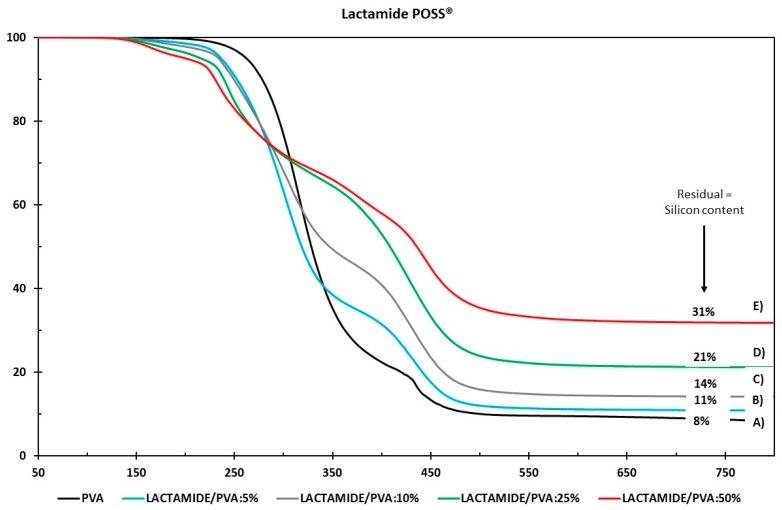
Thermogravimetric analysis of (A) PVA, Lactamide POSS^®^/PVA: (B) 5%; (C) 10%; (D) 25%; (E) 50%.

The thermal stability of the nanocomposite membranes with various nanoparticle loadings was studied by comparing the decomposition onset temperatures presented in Figure 6 and Figure 7 for the Amidino POSS^®^/PVA and Lactamide POSS^®^/PVA systems, respectively. The pure PVA sample shows a major weight loss around 280 °C, attributed to the start of decomposition of the main polymer chain ending around 500 °C. The Amidino POSS^®^/PVA system shows two different major weight losses. The first one lies between 220 and 250 °C, attributed to the decomposition of the NH_2_ groups and the start of the decomposition of the polymer chain, continuing with a second step at 400–450 °C, due to the byproducts generated by PVA during degradation [43,44]. The addition of the nanoparticles decreases this first step, however, the second weight loss, between 400–410 °C, increases with the addition of the nanoparticles. These weight losses correspond to the degradation of the polymer main chain, and the generated byproducts of the PVA. The systems end with different residuals for every sample, increasing with the nanoparticle loading. On the other hand, membranes prepared with Lactamide POSS^®^ nanoparticles present a similar thermal stability as two major weight losses are observed in the range between 400–420 °C. The lactamide groups increase, slightly, the degradation of the polymer chain in this step, to reach a different final residual as well, depending on the concentration of the loading. The number corresponds initially to the residual of the PVA, and then to the increase of residual silicon content by the addition of nanoparticles. The weight loss between 100–200 °C in the samples containing nanoparticles could be due to either condensation of open POSS structures generating water, or due to the release of CO_2_ already reacted with the amine and amidine groups from Amidino POSS^®^ and Lactamide POSS^®^ [45]. These decreased thermal stabilities can be explained by the increase in mobility of the PVA chains in the nanocomposite membranes. The chain transfer reaction will then be promoted, and consequently, the degradation process will be faster with the decomposition taking place at lower temperatures. However, the final complete degradation takes place at higher temperatures by the addition of the nanoparticles, which also change depending on the functionalization, corresponding to the Lactamide POSS^®^ having the highest final degradation temperature. 

#### 3.2.2. Differential Scanning Calorimetry (DSC)

**Figure 8:**
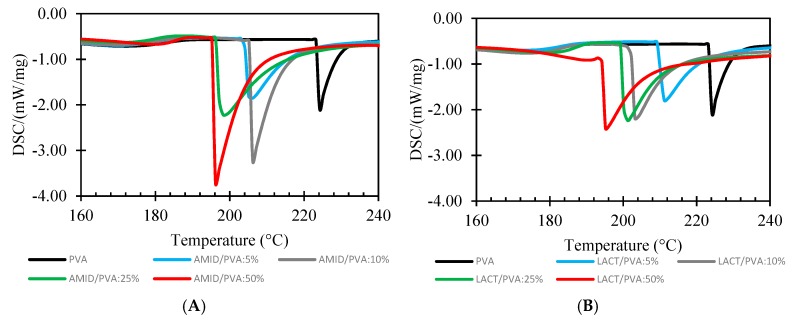
DSC analysis of Amidino POSS^®^/PVA (**A**); Lactamide POSS^®^/PVA (**B**). PVA (black), 5% (blue), 10% (gray), 25% (green), 50% (red).

Figure 8 shows the DSC thermograms for the melting enthalpies for the Amidino POSS^®^/PVA and Lactamide POSS^®^/PVA. The degree of crystallinity ($X_{c}$) is calculated from the ratio $X_{c}=\left(∆H_{f}/∆H_{f^{{}^{\circ}}}\right)$, where $∆H_{f}$ is the melting enthalpy measured, and $∆H_{f^{{}^{\circ}}}$ the 100% crystalline melting enthalpy, respectively. The melting enthalpy ($∆H_{f^{{}^{\circ}}})$ of a 100% crystalline PVA, is taken as 138.6 J/g [39]. 

Table 2 lists the values for the glass transition temperatures T_g_, enthalpy and melting temperature, and degree of crystallinity ($X_{c}$) of the different nanocomposite membranes for the system Amidino POSS^®^/PVA and Lactamide POSS^®^/PVA, respectively. The glass transition increases for a loading of 5%, due to the rigidification of the polymer chain by the addition of the nanoparticles. However, when reaching 10%, the glass transition starts to decrease, due to the increment on the degree of the crystallinity. When reaching 25%, the T_g_ increases further, and at this point, the disturbance of the crystalline regions is higher as more crystalline segments are found—this is detectable by the increment in the degree of crystallinity. The interaction of the nanoparticles with the polymer is visible by the way the polymer chains packed, changing the type of lamella formation, and by the decrease in the melting temperature (T_m_). The melting point (T_m_) of the composite membranes is lower than the virgin PVA polymer membrane. Thus, the melting point of the composite material decreases because of the addition of the POSS^®^ nanoparticles. The decrease in T_m_ with the addition of POSS^®^ indicates that incorporation of POSS^®^ encourages the crystallization process, and results in crystallites with lower thermal stability. This is explained as POSS^®^ acting as a nucleating agent, increasing the degree of crystallinity [31,46]. The obtained numerical values are higher than 100%, because the degree of crystallinity is calculated, dividing the melting enthalpy measured by the 100% crystalline melting enthalpy. More energy is required to melt the crystalline segments formed by the addition of the nanoparticles, thus, the melting enthalpy measured by the addition of the nanoparticles is higher than the pure 100% crystalline polymer.

#### 3.2.3. Dynamic Mechanical Analysis (DMA)

##### Storage Modulus (E’)

Table 3 shows the values of the storage modulus for the two types of nanocomposite membranes. The storage modulus (E’) is equivalent to Young’s modulus of elasticity, which could be an indication of the hardness of the material. This is the response of the sample after receiving the strain, giving the amount of energy required to do so, expressed as modulus [33]. 

The composite membrane shows a typical thermoplastic behavior. The storage modulus is higher for the pure polymer, and it decreases slightly with the increase of nanoparticle loading, corresponding to the primary relaxation associated with the glass–rubber transition of amorphous PVA. Then, when the loading reaches 50%, the E’ modulus increases as compared to the virgin PVA. In this case, this increment can be explained by the crystallization occurring by the addition of POSS^®^ acting as nucleating agent. The difference in the values between the Amidino and Lactamide POSS^®^ can be due to the lactic moieties that are believed to improve compatibility of Lactamide POSS^®^ with PVA, as it contains secondary hydroxyl groups.

##### Tan Delta (Tand)

**Figure 9:**
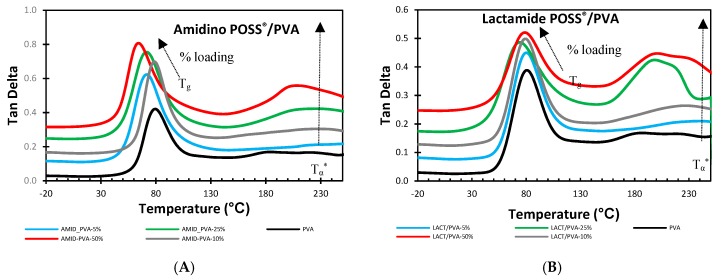
DMA analysis of Amidino POSS^®^/PVA (**A**); Lactamide POSS^®^/PVA (**B**). PVA (black), 5% (blue), 10% (gray), 25% (green), 50% (red). The curves are shifted vertically for clarity.

Figure 9 shows the change in tan delta by the change in the percentage of loading for the two types of nanoparticles, Amidino POSS^®^/PVA, and Lactamide POSS^®^/PVA, respectively. 

The addition of the Amidino POSS^®^ and Lactamide POSS^®^ nanoparticles show that the tan delta gradually decreases by the loading of the nanoparticles. A second peak is found in the region above the T_g_. This peak is also known to be the alpha star transition (T_α_^*^). In semi-crystalline polymers, this transition is associated with the slippage between crystallites [33,47]. When increasing the loading of POSS^®^ nanoparticles, these materials produce a different type of crystalline segment, thus inducing a modification of the tan delta (Tand T_g_) values, and then, an increase of the alpha start transition is visible induced by the movement (slippage) of the crystalline segments. These changes are confirmed in the DSC measurements, where the melting temperature (T_m_) is decreasing as an indication of forming different lamellar conformation. The alpha star transition also increased with the nanoparticles loading, see Table 4. A similar behavior is also observed for the storage modulus (E’), see Table 3. On the other hand, the Lactamide POSS^®^/PVA system shows that the addition of these nanoparticles has a higher effect of interaction since the Tand T_g_ and the alpha star transition (T_α_^*^) are higher in comparison with those obtained in the Amidino POSS^®^/PVA system. Moreover, these interactions are also visible in the degree of crystallinity ($X_{c}$) obtained by DSC, where these values are higher at higher Lactamide POSS^®^ loadings. This improved compatibility was expected as the Lactamide groups can interact better with the PVA polymer chain. Similar behavior is observed for the storage modulus (E’).

### 3.3. Gas Separation Performance of the Nanocomposite Membranes

#### 3.3.1. Effect of Pressure 

**Figure 10:**
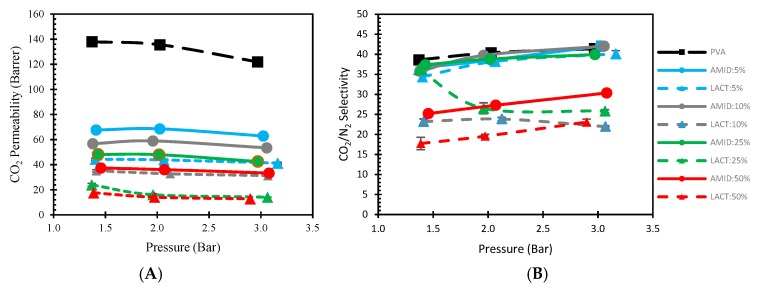
Effect of gas separation performance of nanocomposite membranes by the increment of pressure. Permeability (**A**); Selectivity (**B**). Amidino POSS^®^/PVA (straight line), Lactamide POSS^®^/PVA (dash line), PVA (black), 5% (blue), 10% (gray), 25% (green), 50% (red).

The effect of the feed pressure could have an important significance on the gas separation for facilitated transport membranes. The facilitated transport is based on a reversible reaction of CO_2_, H_2_O, and the fixed carrier. As a general trend, with increasing feed pressure, the concentration of CO_2_ dissolved into the membrane, increases. As the CO_2_ concentration increases, some carriers may be saturated, and fewer sites are available to facilitate the CO_2_ transport, resulting in a decrease of CO_2_ permeance [48,49]. In other words, the CO_2_ permeance may decrease due to a high CO_2_ partial pressure on the feed side saturating the carriers [22,34]. To investigate this behavior of the membranes in the lower feed pressure region, tests were carried out by varying the feed pressure between 1.3 and 3 bars as shown in Figure 10. The performance of the membranes shows a slight decrease in CO_2_ permeability, while the selectivity increases with the pressure, whereas a bigger change is visible in the loading for lactamide 25% between the pressure range 1.3 to 2 bar; this is possibly due to some inaccuracy during measurement. Unlike CO_2_ permeance, N_2_ permeance decreases with increasing feed pressure, which is consistent with the characteristic of ‘‘solution-diffusion’’ mechanism [50], resulting in the little increment in selectivity. The virgin PVA membrane shows the best performance in term of both permeability and selectivity, which is in accordance with our previous research [32]. This trend clearly shows that the membranes do not follow a facilitated transport mechanism, instead, the gas transport is dominated by the solution-diffusion mechanism, and the nanoparticle loading is not contributing with additional CO_2_ transport to the gas separation in the tested pressure range.

#### 3.3.2. Effect of Nanoparticles Structure

**Figure 11:**
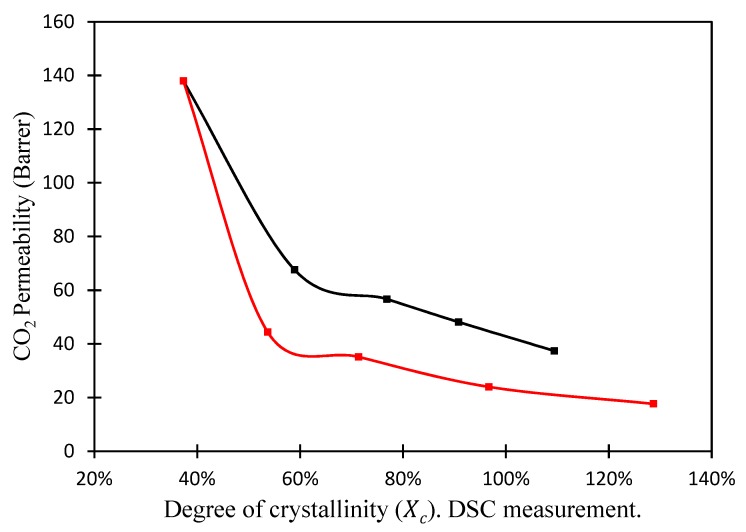
CO_2_ permeability (1.3 bar) of nanocomposite membranes versus degree of crystallinity ($X_{c}$) DSC measurement. Amidino POSS^®^/PVA (black), Lactamide POSS^®^/PVA (red).

By changing the length and the chemical structure of the substituents, POSS^®^ can give different properties to the polymer, like thermal and chemical stability, and acts as a nucleating agent [51,52,53,54]. Figure 11 presents the CO_2_ permeability as function of the degree of crystallinity ($X_{c}$), induced by the addition of the POSS^®^ nanoparticles. The CO_2_ permeability clearly decreases as the degree of crystallinity increases. Moreover, the permeability is always lower when Lactamide POSS^®^ is added to the system. In other words, the higher the degree of crystallinity obtained with the lactamide groups, the lower the gas separation performance. The formation of crystalline segments makes the composite membrane less permeable. It is visible that the structure of the nanoparticle affects the permeance. Both membranes showed lower performance compared to the pure polymer. It seems that the addition of the nanoparticles hindered the CO_2_ gas transport through the membrane. The most significant effect was observed at high Lactamide POSS^®^ loading of nanoparticles. At high loading, more crystalline segments are obtained, as they are gas impermeable, reducing the performance of the membrane.

#### 3.3.3. Effect of Loading Concentration

**Figure 12:**
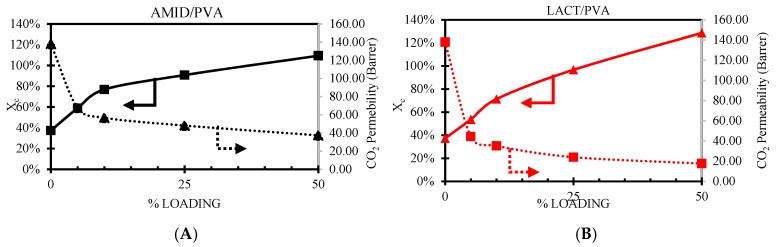
Effect of CO_2_ permeability, dotted line, and degree of crystallinity ($X_{c}$), solid line, versus nanoparticle loading. Amidino POSS^®^/PVA (A), Lactamide POSS^®^/PVA (B).

Figure 12 shows the CO_2_ permeability and the degree of crystallinity ($X_{c}$) follow when increasing the nanoparticle loading. The crystalline regions on the PVA are impermeable to the penetrating molecules [55]. The addition of nanoparticles clearly shows an increment of the degree of crystallinity as the POSS^®^ nanoparticles act as nucleating agent. The Lactamide POSS^®^ group has a stronger interaction with the PVA, enhancing the chain packing order, increasing the degree of crystallinity. On the other hand, the CO_2_ permeability has an opposite behavior, decreasing when increasing the nanoparticles loading. This decrease is reasonable, since these segments are less permeable. The addition of the nanoparticles does not contribute to any facilitated transport mechanism, moreover, it seems to obstruct the space the CO_2_ can use to pass through the composite membrane, resulting in a decrease in CO_2_ permeance. These changes in the gas separation properties could be also due to a decrease in the fractional free volume (FFV), and more research about this is needed. 

## 4. Conclusions

Two different types of POSS^®^ nanoparticles (Amidino POSS^®^ and Lactamide POSS^®^) were used to understand the effect of the functional groups on the interaction with the polymer, and the gas separation performance. SEM pictures showed that a continuous composite PVA layer was formed on the support, and no evidence for pores or defects was observed. The FTIR spectra confirmed the structure of the resultant membranes, by showing the change by the addition of the nanoparticles. DSC studies indicated that the glass transition (T_g_) decreased by the addition of the nanoparticle loading. It indicated an increase of the enthalpy of melting within the increase of the degree of crystallinity ($X_{c}$). The melting temperatures showed a decreasing trend, indicating a different lamellar conformation in the polymer chain. The change in T_g_ was also confirmed by the Tand T_g_ by DMA analysis. The addition of the nanoparticles has an effect on the storage modulus, however, it was not possible to observe any clear trend. A second peak T_α_^*^ (alpha star transition) was found using DMA technique. This alpha transition is known to be the transition where the crystallites slip past one another. The value of this alpha transition increased when increasing the loading. In this sense, the Lactamide POSS^®^ system showed a higher interaction with the polymer, as expected, by having a higher alpha star transition T_α_^*^. The gas separation performance showed a decrease for the CO_2_ permeability, while the selectivity slightly increased with the pressure. This trend shows that the membranes did not follow a facilitated transport mechanism, and the nanoparticle loading did not induce extra CO_2_ transport to the gas separation. The system is primarily dominated by the solution-diffusion mechanism. The degree of crystallinity indicated a strong effect in the gas separation performance of the composite membranes. Finally, the permeability was lower when the Lactamide POSS^®^ group was added to the system. This is due to the higher interaction with the polymer, which increases the degree of crystallinity and reduces the composite membrane permeability.

## Figures and Tables

**Table 1:** Main infrared peaks of the nanocomposite membranes [36,37,38,39,40,41,42].

Frequency (cm^−1^)	Bond Type
3260	O–H stretching (PVA)
2910–2942	CH_2_ asymmetric/symmetric stretching (PVA)
1740, 1265	Residual acetyl group
1615	NH_2_ scissoring
1656	N–H bending from amidine–lactamide
1400	N=N bending (amidine)
1142	PVA crystallites
1100	C–O stretching
1080	Si–O–Si asymmetric stretch
1020	Aliphatic C–N stretching (lactamide)

**Table 2:** Glass transition temperatures, enthalpy and melting temperature, and degree of crystallinity of the different nanocomposite membranes.

Sample	Loading (POSS^®^/PVA Ratio)	T_g_ (°C)	∆H_f_ (J/g)	T_m_ (°C)	$X_{c}$(%)
PVA	0	70.5	51.7	223.4	37.3
Amidino POSS^®^	5	76.9	81.6	205.8	58.9
	10	63.2	106.5	205.7	76.8
	25	53.9	125.8	198.6	90.8
	50	67.0	151.6	195.5	109.4
Lactamide POSS^®^	5	77.8	74.4	211.3	53.7
	10	66.9	98.9	203.4	71.3
	25	55.7	134.0	201.0	96.7
	50	72.4	178.3	185.0	128.6

**Table 3:** Storage modulus (E’) for the different nanocomposite membranes.

		PVA	% Loading							
			Amidino POSS^®^				Lactamide POSS^®^			
			5%	10%	25%	50%	5%	10%	25%	50%
Storage Modulus (E’)	Value (GPa)	8.2	6.7	8.0	6.9	8.3	8.0	7.3	7.4	10.1
	Onset Temperature (°C)	61	51	61	48	46	59	59	46	53

**Table 4:** T_α_^*^ (alpha star transition) & Tand T_g_, for the different nanocomposite membranes.

		PVA	% Loading							
			Amidino POSS^®^				Lactamide POSS^®^			
			5%	10%	25%	50%	5%	10%	25%	50%
T_α_^*^	Absolute Value	0.17	0.14	0.16	0.20	0.26	0.16	0.17	0.23	0.27
Tand T_g_	Absolute Value	0.42	0.54	0.56	0.54	0.50	0.40	0.40	0.34	0.30
	Onset Temperature(°C)	79.4	71.8	78.6	71.3	64.1	80.1	79.2	74.1	78.7
